# A DIseAse MOdule Detection (DIAMOnD) Algorithm Derived from a Systematic Analysis of Connectivity Patterns of Disease Proteins in the Human Interactome

**DOI:** 10.1371/journal.pcbi.1004120

**Published:** 2015-04-08

**Authors:** Susan Dina Ghiassian, Jörg Menche, Albert-László Barabási

**Affiliations:** 1 Center for Complex Networks Research and Department of Physics, Northeastern University, Boston, Massachusetts, United States of America; 2 Center for Cancer Systems Biology (CCSB) and Department of Cancer Biology, Dana-Farber Cancer Institute, Boston, Massachusetts, United States of America; 3 Center for Network Science, Central European University, Budapest, Hungary; 4 Channing Division of Network Medicine, Department of Medicine, Brigham and Women’s Hospital, Harvard Medical School, Boston, Massachusetts, United States of America; University of Chicago, UNITED STATES

## Abstract

The observation that disease associated proteins often interact with each other has fueled the development of network-based approaches to elucidate the molecular mechanisms of human disease. Such approaches build on the assumption that protein interaction networks can be viewed as maps in which diseases can be identified with localized perturbation within a certain neighborhood. The identification of these neighborhoods, or *disease modules*, is therefore a prerequisite of a detailed investigation of a particular pathophenotype. While numerous heuristic methods exist that successfully pinpoint disease associated modules, the basic underlying connectivity patterns remain largely unexplored. In this work we aim to fill this gap by analyzing the network properties of a comprehensive corpus of 70 complex diseases. We find that disease associated proteins do not reside within locally dense communities and instead identify *connectivity significance* as the most predictive quantity. This quantity inspires the design of a novel Disease Module Detection (DIAMOnD) algorithm to identify the full disease module around a set of known disease proteins. We study the performance of the algorithm using well-controlled synthetic data and systematically validate the identified neighborhoods for a large corpus of diseases.

## Author Summary

Diseases are rarely the result of an abnormality in a single gene, but involve a whole cascade of interactions between several cellular processes. To disentangle these complex interactions it is necessary to study genotype-phenotype relationships in the context of protein-protein interaction networks. Our analysis of 70 diseases shows that disease proteins are not randomly scattered within these networks, but agglomerate in specific regions, suggesting the existence of specific *disease modules* for each disease. The identification of these modules is the first step towards elucidating the biological mechanisms of a disease or for a targeted search of drug targets. We present a systematic analysis of the connectivity patterns of disease proteins and determine the most predictive topological property for their identification. This allows us to rationally design a reliable and efficient Disease Module Detection algorithm (DIAMOnD).

## Introduction

**Fig 1:**
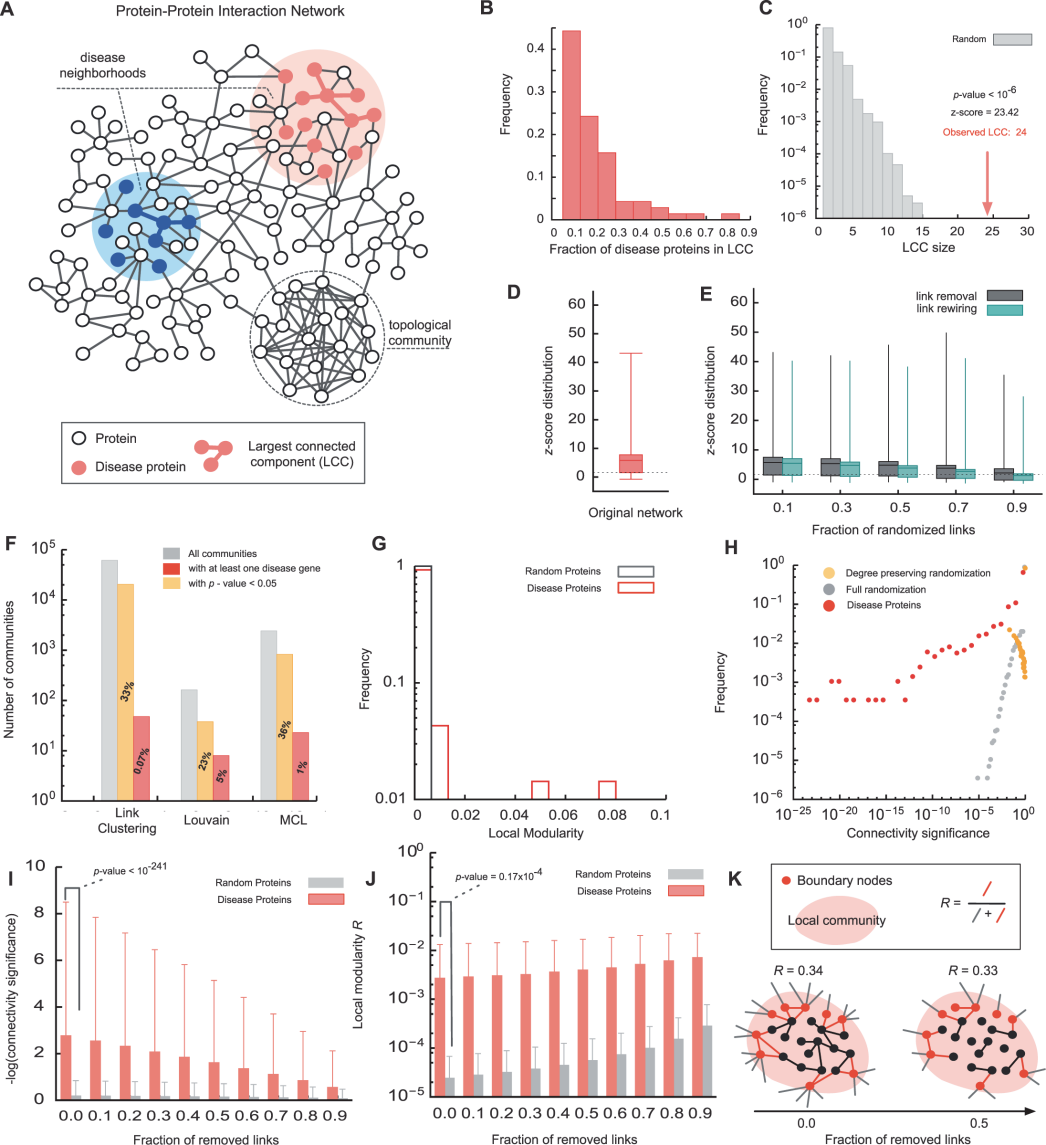
Topological properties of disease proteins within the Interactome. (A) Proteins associated with the same phenotype tend to localize in specific neighborhoods of the Interactome, indicating the approximate location of the corresponding *disease modules*. Topological network communities are highly interconnected groups of nodes. (B) Distribution of the fraction of disease proteins within the largest connected component (LCC) for 70 diseases. Only 10%-30% of the disease proteins are part of the LCC. (C) LCC size of proteins associated with *lysosomal storage disease* compared to random expectation. Out of 45 disease proteins, 24 (53%) are part of the LCC (*z*-score = 23.42, empirical *p*-value < 10^–6^). (D) Significance of the LCC sizes as measured by the *z*-score for all 70 considered diseases. The whiskers indicate the minimum, 25^th^, 50^th^, 75^th^ percentile and maximum across all diseases. Overall, 70% of the diseases show significant clustering (*z*-score>1.6). (E) LCC z-score distribution in noisy networks in which a fraction *f* of all links is randomized by either link removal or rewiring. (F) We applied three representative community detection algorithms to explore the extent to which *topological* modules correspond to *disease* modules. Only 1%-5% of the communities detected by the different methods are significantly enriched with disease proteins, none of which includes a significant fraction of all disease proteins. (G) Comparison of the distribution of the local modularity *R* for disease proteins and proteins randomly selected from the Interactome. (H) Distribution of the connectivity significance of disease proteins and randomly selected proteins. (I) Connectivity significance of disease proteins as a function of the fraction *f* of links removed from the network. The red bars denote the mean and the standard deviation as measured across 70 diseases, yellow bars show random expectation obtained from the same number of randomly distributed genes. (J) Local modularity of disease proteins and randomly selected proteins when a fraction *f* of the links is removed from the network. (K) Illustration of the local modularity *R*.

In the recent years, there is increasing evidence that proteins associated with a particular disease have distinct interactions within the *Human Interactome*, representing the cellular network of all physical molecular interactions [1–7]. The pathobiological properties of a disease and its clinical manifestations can be linked to perturbations within these disease neighborhoods, or *disease modules* [8]. With recent advances in genome-wide disease gene association [9] and high-throughput Interactome mapping [10] we can already pinpoint the approximate location for some disease modules (Fig. 1A). For many diseases, however, a considerable fraction of their disease associations remain unknown [11]. In this paper, we propose a network-based methodology to uncover the disease module associated with a particular phenotype. The algorithm is based on a systematic analysis of the network properties of known disease proteins across 70 diseases, revealing that instead of connection *density* the connectivity *significance* is the most predictive quantity characterizing their interaction patterns. This quantity allows us to systematically explore the local network neighborhood around a given set of known disease proteins, helping us identifying promising new disease protein candidates.

## Results

### Interaction patterns of disease proteins within the Interactome

We started by compiling a comprehensive list of experimentally documented molecular interactions in human cells as described in [12] (see Methods). We also curated a list of 70 well-characterized complex diseases (Table 1) and their known associated proteins from OMIM [13] and GWAS [9] (see Methods). In total, we obtained 141,296 interactions between 13,460 proteins, 1,531 of which are associated with one or more diseases. Examining the subgraphs consisting of proteins associated with the same disease, we found that the largest connected component (LCC) typically contains only 10%-30% of the disease proteins (Fig. 1B). This surprisingly low fraction has been shown to be a direct consequence of the incompleteness of currently available interactome maps [12]. Yet, despite this apparent scattering, the observed agglomeration is typically still higher than expected for randomly distributed proteins (Fig. 1C). The LCCs of 49 (out of 70) diseases are significantly larger (*z*-score > 1.6) than random expectation (Fig. 1D, Table 1). To explore the possible influence of noise in the underlying Interactome on the observed clustering we repeated the analysis on perturbed networks with varying degrees of noise and incompleteness (see Methods). Fig. 1E shows that ∼50% of all diseases exhibit significant LCCs even after removing or randomizing up to 90% of the links in the network, indicating that the finding that disease proteins tend to reside in specific network neighborhood is remarkably robust.

**Table 1:** List of the 70 considered diseases.

Disease	#genes (LCC)	*z*-score	*p*-value	Disease	#genes (LCC)	*z*-score	*p*-value
adrenal gland diseases	18 (5)	8.13	3.09e-4	glomerulonephritis	18 (3)	3.83	0.02
alzheimer disease	29 (6)	6.55	8.13e-4	gout	13 (1)	-0.33	1.0
Amino acid metabolism inborn errors	52 (13)	10.27	2.5e-5	graves disease	13 (2)	2.57	0.11
amyotrophic lateral sclerosis	21 (2)	1.33	0.25	head and neck neoplasms	35 (4)	2.94	0.03
anemia aplastic	21 (9)	14.49	2.12e-4	hypothalamic diseases	23 (2)	1.15	0.29
anemia hemolytic	29 (7)	8	2.12e-4	leukemia b-cell	17 (2)	1.82	0.18
aneurysm	15 (4)	7.22	1.15e-3	leukemia myeloid	43 (17)	16.67	0.0
arrhythmias cardiac	30 (5)	4.91	3.87e-3	lipid metabolism disorders	50 (4)	11.62	2e-6
arthritis rheumatoid	42 (9)	7.95	2.53e-4	liver cirrhosis	24 (2)	1.07	0.32
asthma	37 (3)	1.53	0.12	liver cirrhosis biliary	23 (2)	1.15	0.29
arterial occlusive diseases	44 (4)	2.19	0.06	Lung diseases obstructive	40 (4)	2.49	0.04
arteriosclerosis	38 (4)	2.66	0.03	lupus erythematosus	75 (7)	1.26	0.13
basal ganglia diseases	45 (8)	6.39	1.13e-3	lymphoma	24 (2)	1.07	0.32
behcet syndrome	13 (2)	2.57	0.11	lysosomal sorage diseases	45 (24)	23.42	0.0
bile duct diseases	31 (2)	0.6	0.46	mascular degeneration	44 (8)	6.53	9.36e-4
blood coagulation disorders	40 (25)	26.91	0.0	metabolic syndrome x	14 (3)	5.06	8.52e-3
blood platelet disorders	26 (7)	8.82	1.03e-4	motor neuron disease	31 (2)	0.6	0.46
breast neoplasms	40 (18)	18.74	0.0	multiple sclerosis	69 (11)	5.87	1.89e-3
carbohydrate metabolism inborn errors	77 (11)	4.94	4.31e-3	muscular sydtrophies	36 (12)	12.86	2e-6
carcinoma renal cell	18 (3)	3.84	0.02	mycobacterium infections	22 (4)	4.86	4.91e-3
cardiomyopathies	50 (12)	9.65	6.6e-5	myeloproliferative disorders	19 (6)	9.76	6.1e-5
cardiomyopathy hypertrophic	22 (4)	1.86	4.96e-3	metabolic and nutritional diseases	599 (270)	4.04	2e-6
celiac disease	36 (2)	0.34	0.56	peroxisomal disorders	20 (17)	30.86	0.0
cerebellar ataxia	30 (2)	0.66	0.44	psoriasis	54 (5)	2.47	0.04
cerebrovascular disorders	47 (4)	1.98	0.07	purine-pyrimidine metabolism inborn errors	16 (2)	1.98	0.16
charcot-marie-tooth disease	27 (5)	5.46	2.32e-3	renal tubular transport inborn errors	34 (3)	1.74	0.10
colitis ulcerative	56 (4)	1.44	0.12	sarcoma	25 (7)	9.13	8.4e-5
colorectal neoplasms	42 (16)	15.83	0.0	spastic paraplegia hereditary	20 (1)	-0.51	1.0
coronary artery disease	31 (2)	0.6	0.46	spinocerebellar ataxias	28 (2)	0.78	0.40
crohn disease	72 (10)	4.82	4.91e-3	spinocerebellar degenerations	30 (2)	0.65	0.44
death sudden	19 (1)	-0.49	1.0	spondylarthropathies	18 (4)	5.99	2.26e-3
diabetes mellitus type 2	73 (9)	4.03	9.83e-3	taupathies	35 (9)	9.32	5.6e-5
dwarfism	26 (3)	2.5	0.05	uveal diseases	17 (3)	4.07	0.01
esophageal diseases	24 (3)	2.76	0.04	varicose veins	20 (1)	-0.51	1.0
exophthalmos	13 (2)	1.58	0.11	vasculitis	15 (2)	2.16	0.14

List of the 70 diseases considered in this study, together with their respective number of associated genes and the size of their largest connected component (LCC) on the Interactome, as well as its significance compared to randomly selected genes as given by the *z*-score and the empirical *p*-value obtained from 10^6^ simulations.

From a network science perspective, the task of identifying these disease neighborhoods can be considered a *community detection* problem. Numerous algorithms [14–23] define a community as a locally dense subgraph in a network (Fig. 1A). In order to evaluate the extent to which such topological community detection algorithms can be used to predict disease modules, we chose three representative, methodologically distinct algorithms that have been successfully applied to identify communities of functionally related proteins (*functional* modules) in protein interaction networks: *(i)* A link community algorithm [14], which is based on link-similarities and can also capture hierarchical communities, *(ii)* the Louvain method, which maximizes a global modularity function [21], and *(iii)* the Markov Cluster Algorithm (MCL), which detects dense regions based on random flow [24]. Each of these methods identifies a large number of communities within the Interactome (Figs. 1F & S1A-C). In order to evaluate whether some of these communities may be candidates for specific disease modules, we determined their enrichment with known disease proteins. We found that only between ∼1%-5% of the communities detected by the different methods are significantly enriched (*p*-value < 0.05, Fisher’s exact test) with any set of disease proteins (Fig. 1F). Conversely, only 15% of the diseases have any significantly enriched community. As these significantly enriched communities cover only ∼15%-38% of all proteins associated with the respective disease, we were unable to assign for any of these diseases a single connected disease module (S1 Fig. D-F).

These results suggest that while topological communities may often represent meaningful *functional modules [25],* they are not able to capture *disease modules*. One possible reason for this may be that disease proteins do not constitute particularly dense subgraphs. To further quantify this, we consider the modularity parameter *R* [23], a key measure used in community detection, where *R* = 1 corresponds to perfect modularity and *R*∼0 to randomly assigned communities (see Materials & Methods and Fig. 1K). If we consider the known disease associated proteins as communities, we find that *R*<0.01 for 97% of the diseases, with no disease exceeding *R*>0.07 (Fig. 1G). While these values are still significantly different from random expectation *R*∼0, the communities resulting from optimizing *R* are unlikely to represent meaningful disease modules.

Yet, disease proteins *do* exhibit distinct and predictive connectivity patterns that can be captured and exploited if we evaluate the *significance* of their connections instead of their density. Consider a network of *N* proteins containing a relatively small number (*s*_*0*_) of seed proteins associated with a particular disease. For randomly scattered seed proteins, the probability that a protein with a total of *k* links has exactly *k*_*s*_ links to seed proteins is given by the hypergeometric distribution:

$$p(k,k_{s})=\frac{\left(\begin{array}{c} s_{0}\\ k_{s} \end{array}\right)\left(\begin{array}{c} N-s_{0}\\ k-k_{s} \end{array}\right)}{\left(\begin{array}{c} N\\ k \end{array}\right)}$$ (1)

To evaluate whether a certain protein has more connections to seed proteins than expected under this null hypothesis, we calculate the *connectivity p*-value, i.e. the cumulative probability for the observed or any higher number of connections:

$$p-\text{value}(k,k_{s})=\sum_{k_{i}=k_{s}}^{k}p(k,k_{i})$$ (2)

The use of the *significance* of the number of connections instead of their absolute number reduces the spurious detection of high-degree proteins. Fig. 1H shows that the connectivity *p*-values within the sets of known disease proteins are very significantly (*p*-value < 10^–241^, Kolmogorov-Smirnov test) shifted towards smaller values when compared to the distributions expected for randomly scattered proteins. For example, the randomization procedure never yields connectivity significance values smaller than 10^–5^, while 60% of the disease proteins have a connectivity significance smaller than this value, some as small as 10^–23^.

Taken together, these results show that disease proteins exhibit distinct interaction patterns among each other that suggest the existence of specific disease modules within the Interactome. Yet, these modules apparently do not coincide with topological communities of densely interconnected proteins. In principle, this discrepancy could be either a mere consequence of incomplete Interactome and gene-disease association data [5,10,26], or reflect an inherent fundamental difference between disease and topological modules. To investigate this question, we compared the behavior of the two relevant measures, local modularity and connectivity significance, for different levels of completeness of the underlying network. Fig. 1I shows that the connectivity significance of disease genes slowly drops as more and more links are removed. Conversely, this trend indicates that the predictive power of the connectivity significance should continuously increase as the Interactome becomes more and more complete. For the local modularity measure, however, we see a very different behavior. Fig. 1J shows that the modularity remains roughly constant as the network completeness decreases or even slightly increases, similar to the behavior observed for random expectation. The reason for this somewhat unintuitive behavior is that random removal affects links between disease proteins to the same extent as links to other proteins, thereby leaving their relative relationship, on average, unchanged (Fig. 1K). We therefore expect that with increasing network completeness, the local modularity among disease proteins will not significantly increase. These results suggest that topological communities are not able to significantly capture disease proteins, regardless of the level of network completeness. Connectivity significance, on the other hand, captures the interaction patterns between disease proteins more and more distinctively as the network approaches the complete network.

### The DIAMOnD algorithm

**Fig 2:**
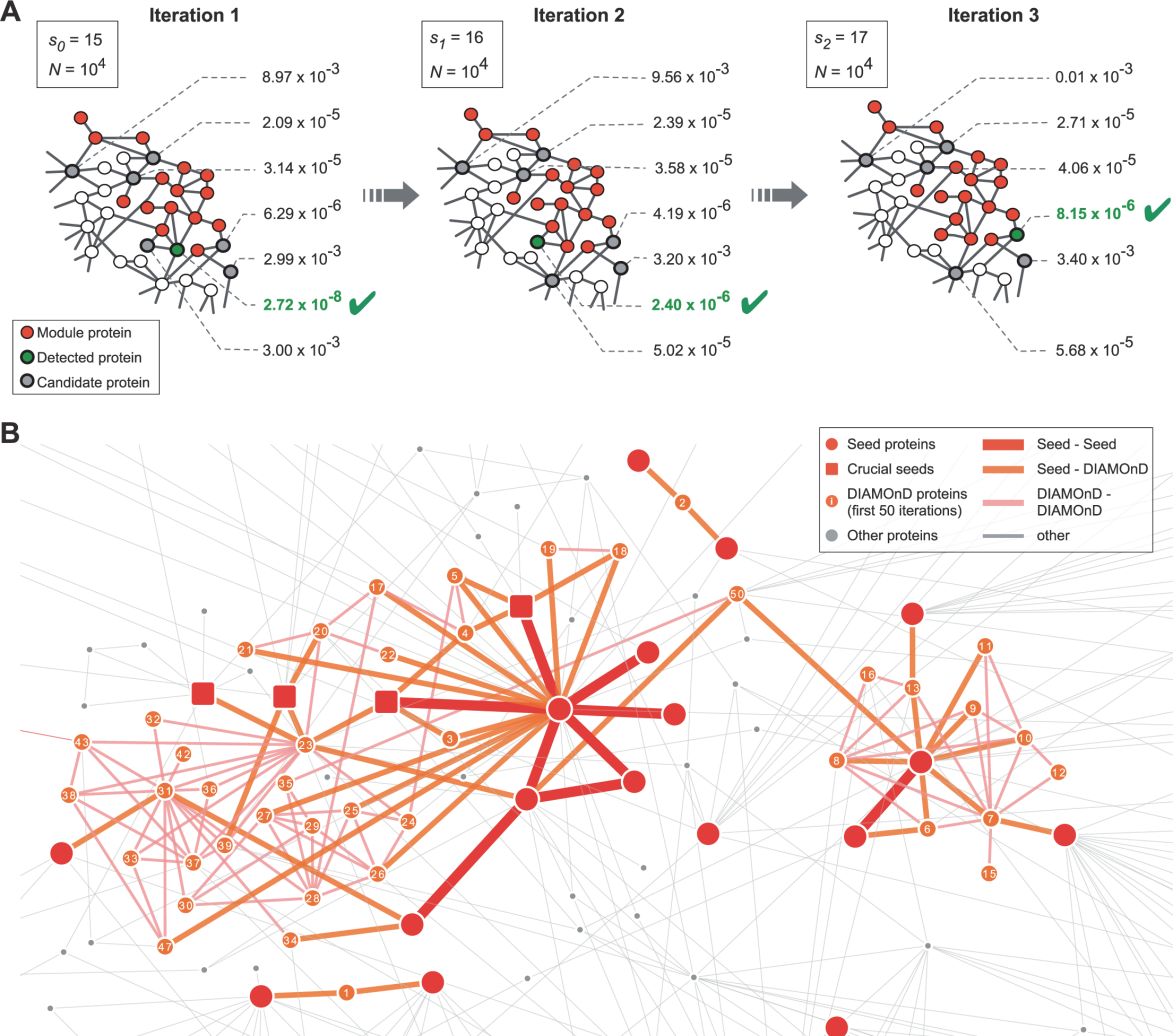
The DIAMOnD algorithm. (A) At each step of the iterative algorithm, the *connectivity significance* of all immediate neighbors of disease proteins is calculated. Next, the most significantly connected node (lowest *p*-value) is integrated into the module, thus expanding the module by one node per iteration step. (B) Subgraph of the Interactome highlighting the seed proteins for *macular degeneration* and the first 50 corresponding DIAMOnD proteins. In the beginning, two separate clusters grow independently until they merge at iteration step 50. Note that DIAMOnD also proposes proteins that do not have direct connections to seed proteins, e.g. at iteration steps 12 and 15. The squares mark seed proteins whose removal leads to large differences in the resulting DIAMOnD modules. The three leftmost squares, for example, enable the identification of a protein at iteration step 23, which in turn triggers the inclusion of the cluster of proteins depicted underneath, which would be absent otherwise.

Building on the observation that the connectivity significance is highly distinctive for *known* disease proteins, we propose the following algorithm to infer yet *unknown* disease proteins (Fig. 2A), and hence to identify the respective disease module:

- The connectivity significance (2) is determined for all proteins connected to any of the *s*_0_ seed proteins.
- The proteins are ranked according to their respective *p*-values.
- The protein with the highest rank (i.e. lowest *p*-value) is added to the set of seed nodes, increasing their number from *s*_*0*_ →*s*_*1*_ = *s*_*0*_+1.
- Steps *(i)-(iii)* are repeated with the expanded set of seed proteins, pulling in one protein at a time into the growing disease module.

The procedure (i)-(iv) can be continued until the module spans across the entire network. The order in which the proteins are being pulled into the module reflects their topological relevance to the disease, resulting in a ranking of all proteins. Fig. 2B shows a subgraph of the Interactome highlighting the seed proteins associated with macular degeneration and the first 50 DIAMOnD genes.

Calculating tens to hundreds of *p*-values at each iteration is computationally expensive; therefore we have implemented an efficient calculation to reduce the execution time (see Materials & Methods). Furthermore, as detailed below, the algorithm can be easily adapted to incorporate additional features, in particular weighted links and/or protein associations.

### Synthetic modules

In order to systematically evaluate the performance of DIAMOnD we first used a well-controlled test scenario by constructing synthetic modules of proteins within the Interactome. We analyzed the extent to which DIAMOnD can recover the full module if we remove the disease association from a certain fraction of proteins, thus obtaining a seed cluster that is no longer fully connected. There are many different possibilities to construct a connected set of nodes in a network, generally leading to modules with different topological properties. We implemented two different methods:

- *Shell-modules*: We randomly selected one node from the network and add all its first and second neighbors to the module (S2 Fig. A). Depending on the particular starting node, the constructed module may vary in size (S2 Fig. B). Most diseases in our curated corpus have between 50 and 150 currently identified disease proteins. Assuming that these represent only 30%-50% of all associated proteins, we chose 200 as the putative size of complete disease modules within the Interactome.
- C*onnectivity significance modules*: We started from a randomly selected node and iteratively add the most significantly connected node to the module until its size reaches 200 nodes. This process produces modules with topological properties similar to those observed for real diseases.

### Estimating the recovery rate

**Fig 3:**
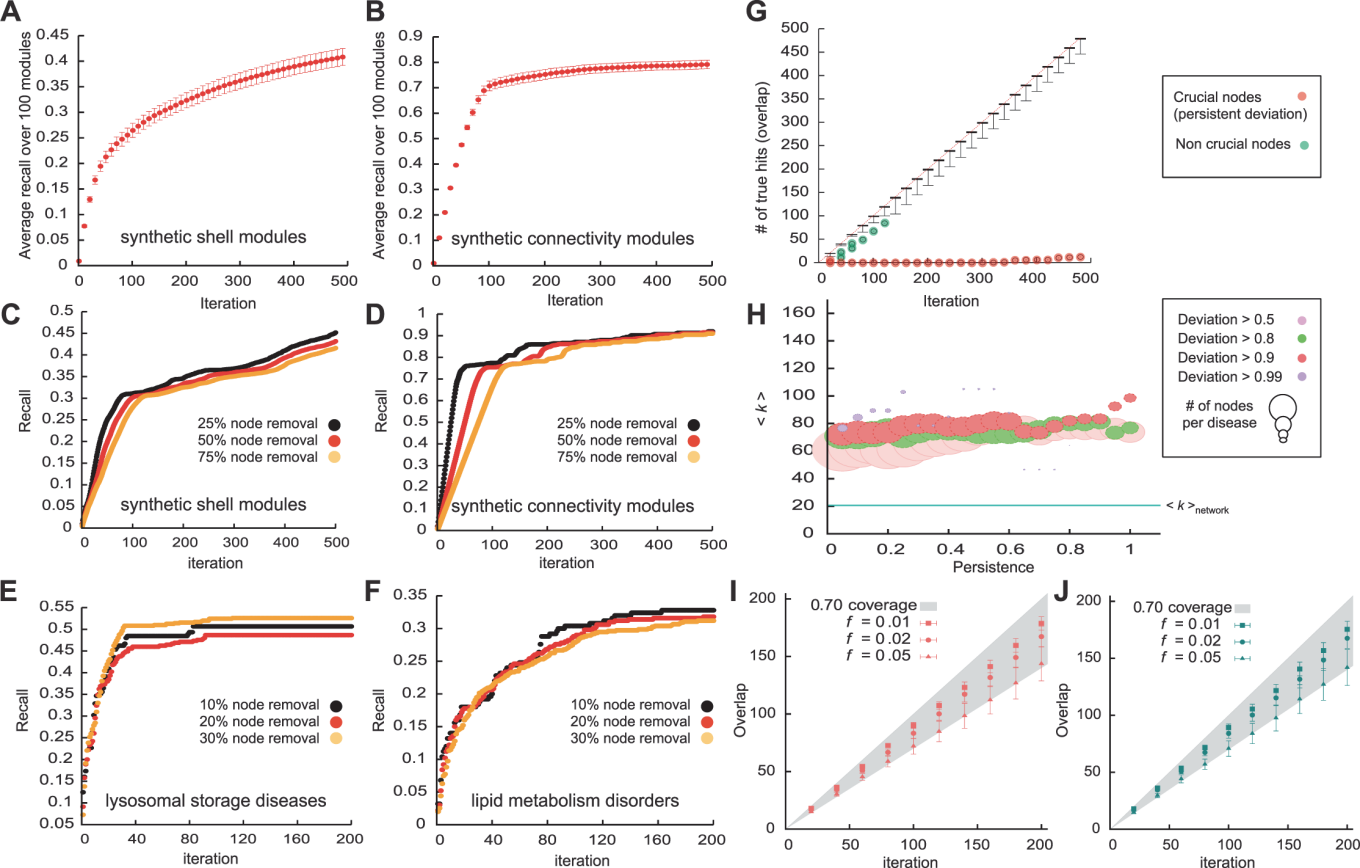
Performance evaluation of DIAMOnD. We use two different methods to construct synthetic modules (*shells* and *connectivity* modules). (A, B) Recovery rate of the DIAMOnD algorithm when removing 50% of seed nodes from *shells* (A) and *connectivity* synthetic modules (B), respectively. The recovery rate in synthetic modules is roughly independent of the module incompleteness. (C, D) Recovery rate when 25%, 50% and 75% of the nodes are removed from *shells* and *connectivity* modules. (E, F) Recovery rate when 10%, 20% and 30% of the nodes are removed from the disease proteins of *lysosomal storage diseases* and *lipid metabolism disorders*. (G) Robustness of the DIAMOnD algorithm towards small variations in the starting seed proteins (*N*-1 analysis). While most nodes influence the outcome very little, there are a few nodes whose removal results in a large deviation from the original outcome. This deviation may either persist across iterations (red data points) or disappear after a few iterations (green). (H) Crucial nodes are characterized by a 3–4 times higher degree. (I) DIAMOnD robustness towards random link removal from the Interactome. We identified the DIAMOnD proteins for 70 diseases in the original Interactome as well as in perturbed networks with varying fractions *f* of randomly removed links. Data points and bars represent the median and median absolute deviation of the overlap (number of common proteins) between original and randomized DIAMOnD sets across 70 diseases as a function of the iteration step. (J) Same as (I), but for perturbed networks in which varying fractions *f* of all links have been randomly rewired.

For each initially connected synthetic module, we randomly removed a certain fraction (25%, 50% and 75%) of the nodes and use the remaining nodes as seed proteins for DIAMOnD. Fig. 3A and 3B show the fraction of recaptured initial seed nodes (recall) as a function of the number of iterations of the algorithm for 50% of the module removed. As expected, the highest rate of true positives is achieved in early iterations, so the highest ranked proteins are most likely to be part of the original full module.

In both *shell* and *connectivity* modules, we find that the total recall of the removed nodes is relatively insensitive to the incompleteness of the seed set, i.e. the fraction of removed seed nodes (Fig. 3C,D). The observation that a similar number of proteins can be recalled from a 25% subset of the full module and from a 75% subset can be used to address a critical limitation of prioritization methods that only provide a ranking of all proteins, yet offer no objective criterion for the total number of biologically relevant proteins. Indeed, estimating the true positive rate is inherently difficult as the true set of proteins is by definition unknown. However, since the recall of DIAMOnD does not depend on the unknown total number of disease proteins, we can estimate it by further pruning a given incomplete set of known disease proteins. We tested this procedure on our set of 70 diseases by removing 10%, 20% and 30% of the respective known disease proteins, see Fig. 3E,F for two examples, *blood coagulation* and *lipid metabolism disorders*, respectively. Generally, the recall is found to be higher when disease associations are preferably removed from proteins that are part of the original LCC.

### Analyzing the sensitivity towards perturbations

Both the network data and the disease associations are inherently noisy and expected to contain a considerable number of false positives. The similar recall from different levels of seed protein incompleteness suggests, however, that collectively the seed proteins and their interactions provide sufficient predictive power to yield robust predictions. In order to evaluate how sensitive the DIAMOnD outcome is with respect to variations in the set of seed genes, we performed an *N*-1 analysis: We modified the initial seed protein set by removing one of the *s*_*0*_ proteins at a time, resulting in *s*_*0*_ different DIAMOnD sets. Comparing the resulting sets of DIAMOnD proteins to the original predictions obtained from the full seed set, we find that the methodology is very robust, yielding overlaps close to 100% in most cases. Individually, most seed proteins can be removed without considerably changing the resulting DIAMOnD proteins. There are, however, typically a small number of nodes whose removal results in a drastic change of the final outcome (Figs. 2B and 3G). The deviation caused by a specific node removal may occur in the initial iterations and disappear over the long run (Fig. 3G, green data points) or persist across all iterations (red data points). These latter nodes are therefore more important for the integrity of the seed set. Fig. 3H shows the degree of the nodes that cause deviations of different persistence (see Materials & Methods). Crucial nodes with high persistence are characterized by a high degree (generally several fold increase compared to both average degree of the network, <*k*> = 20.7, and average degree of the disease proteins, <*k*_disease_> = 28.9). Interestingly, we further observe that crucial nodes whose removal will be most destructive are generally not part of the largest connected component of the initial seed set. Instead, the *disease modules* are robust towards removing disease proteins from the LCC, as these proteins will be recovered early on due to their significant connectivity.

Similar results are obtained when noise is introduced in the underlying network (see Materials & Methods for details). Fig. 3I and 3J show that, regardless of the method we choose to add the noisiness to the network, small variations ∼1% of all links in the Interactome have almost no effect on the obtained DIAMOnD genes. Up to 5% of the Interactome can be completely randomized, while still retrieving more than 70% of the original set of DIAMOnD genes for more than half of all diseases.

### Validating disease modules

**Fig 4:**
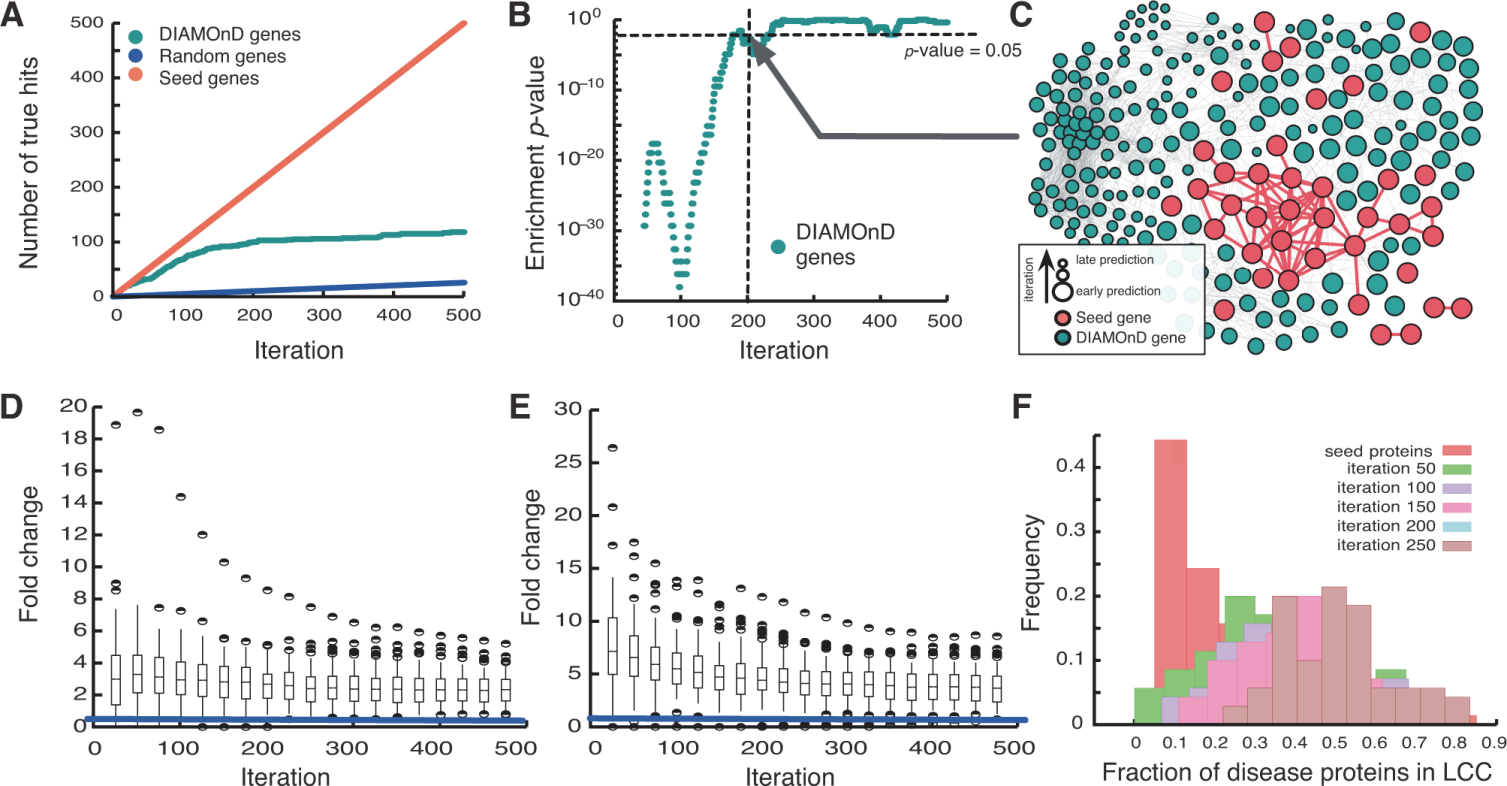
Biological evaluation of DIAMOnD. (A) Validation of the DIAMOnD genes based on GeneOntology terms (see Materials & Methods). (B) The significance of the similarity between DIAMOnD genes and seed genes suggests a cutoff of ∼200 DIAMOnD genes. (C) Network representation of the *lysosomal storage diseases* module. (D,E) Summary of the validation for all 70 disease modules based on GeneOntology (D) and biological pathways (E). (F) Fraction of seed proteins that are contained in the LCC of the DIAMOnD module for varying iteration steps. The distributions show the values obtained from 70 diseases. By introducing DIAMOnD proteins, previously disconnected seed proteins become part of the LCC.

Next we explore the performance of DIAMOnD on 70 real diseases. Since the full set of disease proteins is, by definition, unknown, we cannot assess the performance directly in terms of true positives/negatives. We therefore use publicly available gene annotation data, GeneOntology [27] and biological pathways from MSigDB [28] to validate the DIAMOnD disease modules: For each disease we determine a reference set of all significantly enriched GO-terms and pathways within the set of seed proteins. We then compare the respective annotations of each DIAMOnD gene to this reference set, assuming that proteins with annotations similar to the ones of the seed genes are more likely to be disease associated as well [1,29–32] (see Materials & Methods for details). Fig. 4A,B offers examples for the validation according to pathway similarity for *lysosomal storage diseases*. The first ∼200 DIAMOnD genes are found to participate in important seed pathways at a rate similar to the one within the seed proteins themselves and significantly higher than random expectation. In total, 58 out of 70 disease modules can be validated by either GO terms or pathways, 46 by both. Fig. 4D,E summarizes the validation of the disease modules for all 70 diseases. The majority of the detected modules perform several times better than random expectation, in particular in the first 50–100 iterations.

Depending on the specific application, the main interest of applying DIAMOnD could lie either in selecting a small number of most promising disease protein candidates, or in obtaining a larger set of proteins to explore the molecular disease mechanisms in a broader context. For the former case, DIAMOnD directly offers a ranked list of candidates. The latter approach, however, requires an additional criterion to define the boundary of the disease module, i.e. a threshold for the total number of proteins to be considered. This threshold can be chosen by using either *(i)* topological or *(ii)* biological properties of the agglomerated proteins.

*(i)* The connectivity *p*-values cannot be used directly to define a topological threshold. The reason is that the module grows at each iteration step, i.e. the number of seed genes *s* on which the *p*-value in Eqs. (1) and (2) is based, also increases. Since larger sets can produce smaller *p*-values, the absolute significance values obtained at different iteration steps cannot be compared to each other. However, our analysis suggests and alternative approach to define a topological threshold: As discussed above, the recall of the DIAMOnD algorithm does not depend sensitively on the initial level of completeness (Fig. 3C-F). Hence, the true positive rate can be estimated by removing varying fractions of seed proteins. For *lysosomal storage disorders*, for example we find an estimated recall of ∼50% at iteration 40 (Fig. 3E). After 40 iterations, the recall saturates and reaches a plateau, indicating that thereafter only few DIAMOnD proteins are expected to be truly disease associated. This saturation point may therefore be used as a threshold for the total number of DIAMOnD genes to consider.

*(ii)* A biological criterion for the threshold can be obtained from the validation according to Fig. 4A,B. The number of DIAMOnD proteins with direct biological evidence reaches a plateau at ∼200 iteration steps, suggesting this as the maximal number that should be considered. A more stringent criterion is to use the significance of the enrichment (see Materials & Methods). The enrichment is typically strongest within the highest ranked DIAMOnD proteins and decreases with increasing iteration steps. For *lysosomal storage diseases*, for example, we find that the first 200 DIAMOnD proteins are similarly significantly enriched as the seed proteins (Fig. 4B). The largest connected component of the seed proteins aloneconsists of 24 (out of 45) proteins. When 200 DIAMOnD proteins are added, the largest connected component of the resulting module integrates 11 additional, previously disconnected seed proteins, resulting in a module consisting of 234 proteins (Fig. 4C). Fig. 4F shows the distribution of the fraction of integrated seed proteins across 70 diseases for several iterations. We find that with increasing number of DIAMOnD genes more and more disconnected seed proteins are integrated into the module, thus allowing for an integrated analysis of their molecular mechanism.

### Comparison with existing methods

In recent years, a number of disease protein prioritization methods [24,29,33–36] have been developed that can in principle be used to identify disease modules. To evaluate the relative performance of DIAMOnD, we implemented a random walk based algorithm (RW) [35] that was shown to outperform other methods and may therefore serve as a reference [29].

**Fig 5:**
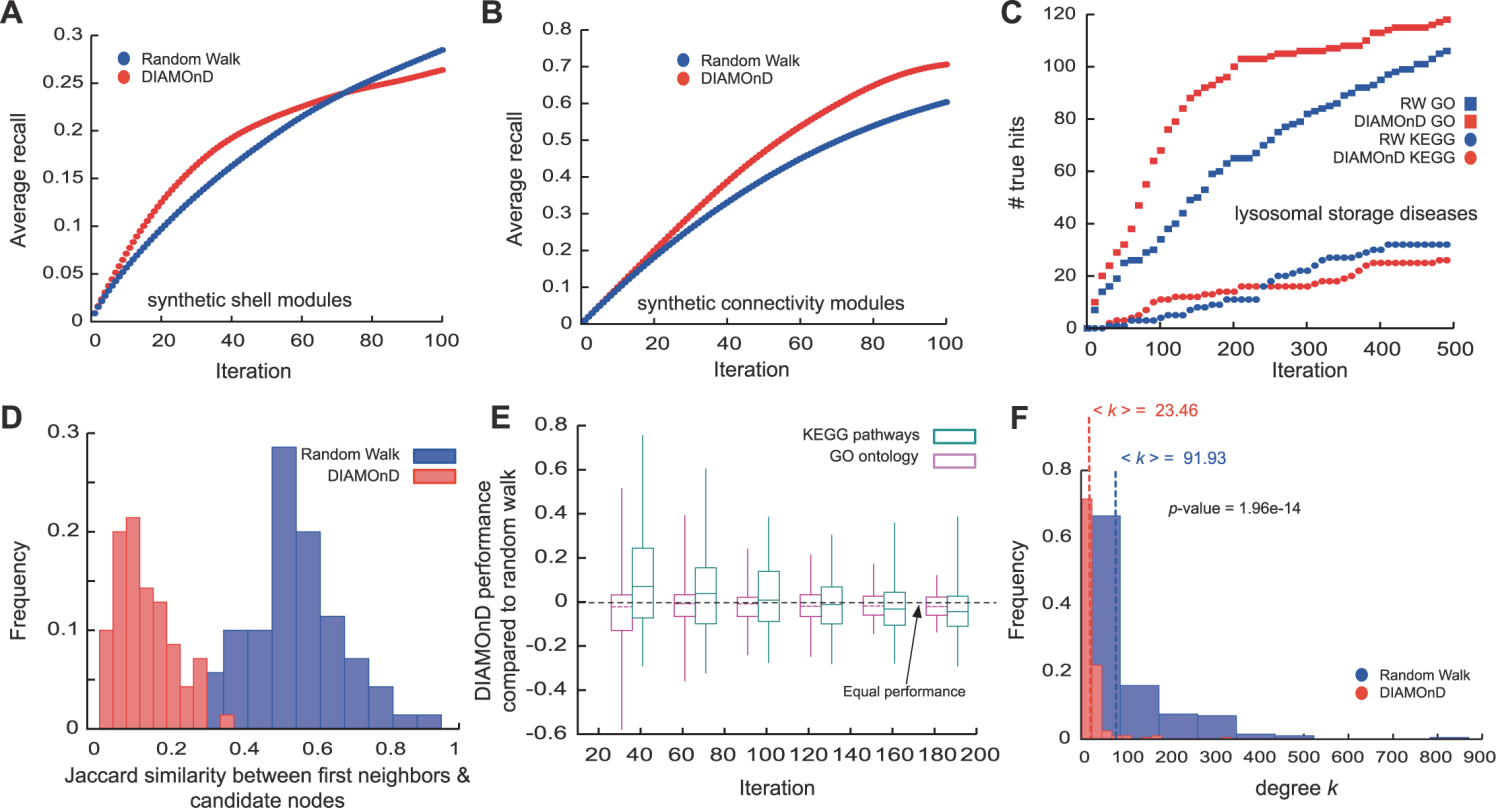
Comparison between DIAMOnD and Random Walk (RW). (A,B) Average recovery rates of DIAMOnD and the reference RW algorithm when removing 50% (100 nodes) of 100 generated *shells* (A) and *connectivity* (B) modules. (C) Comparison of the biological evidence for proteins identified by DIAMOnD and RW for *lysosomal storage diseases*. (D) Overlap between identified proteins and immediate neighbors of seed proteins. In contrast to RW, DIAMOnD includes a considerable number of proteins without first-order interactions to seed genes. (E) Comparison of the performance of DIAMOnD and RW across 70 diseases with respect to non-specific disease data. (F) Degree distributions of the identified proteins. DIAMonD proteins are characterized by the absence of hubs.

Fig. 5A,B summarizes the results of the comparison between DIAMOnD and RW on the synthetic modules. As we removed the attribute from half of the module nodes (about 100 nodes), iteration step 100 is a reasonable point of comparison. For both types of synthetic modules we find that DIAMOnD has a higher recovery in the top 100 predictions, whereas RW captures more true hits in its late predictions. In most cases DIAMOnD is able to identify removed nodes in the early iterations until the recovery rate saturates (Fig. 5A). A higher initial slope corresponds to higher precision, i.e. a higher ratio of true positives TP/(TP+FP). DIAMOnD shows higher precision and sensitivity (recall) in the initial iterations whereas RW performs better at later iterations once DIAMOnD saturated. In the context of disease protein identification, a high quality detection of fewer proteins with few false positives is generally more desirable than low quality detection of hundreds of proteins.

We also compared the predictions of DIAMOnD and RW for each of the 70 real disease modules, as illustrated in Fig. 5C for *lysosomal storage* diseases. In general, DIAMOnD offers several conceptual and practical advantages compared to previous methods: (a) Many methods like RW preferentially select proteins from the immediate neighborhood of the seed proteins. Surprisingly, we find that a considerable fraction of the DIAMOnD proteins do not directly interact with seed genes (Figs. 2B and 5D). DIAMOnD thereby offers disease-relevant candidates beyond first-order protein interactions. (b) Physically interacting proteins often share functional annotations and pathways [10,25]. As a consequence, methods like RW are expected to perform well on generic validation data. In our comprehensive analysis across 70 diseases we are limited to such generic validation data and hence observe a comparable performance when GO term similarity is used as reference. Yet, we find that when we use pathways DIAMOnD outperforms RW (Fig. 5E). Furthermore, a more focused study on a single disease that used a variety of disease-specific data, e.g. from GWAS, microarray experiments and comorbidity analysis, has experimentally confirmed the specific disease-relevance of the DIAMOnD genes and significant outperformance of DIAMOnD over RW [37]. (c) By design, DIAMOnD avoids the selection of spurious high degree nodes. Consequently, the resulting modules are generally characterized by the absence of hubs. RW proteins, in contrast, have 2–3 times higher average degree (Fig. 5F). (d) The recall rate of the DIAMOnD algorithm is roughly independent of the level of incompleteness in the seed genes. It therefore allows us to estimate the number of biologically relevant predictions (Fig. 3C-F). In contrast, methodologies like RW solely provide a ranking, without predicting the total number of the most probable candidates. (e) DIAMOnD shows a significantly higher recall in the early iterations compared to RW, thereby providing higher confidence candidates early on. (e) As we discuss below, the DIAMOnD algorithm can be fine-tuned for specific applications, for example by giving varying weights to the initial seed genes.

### Extending the basic DIAMOnD algorithm

**Fig 6:**
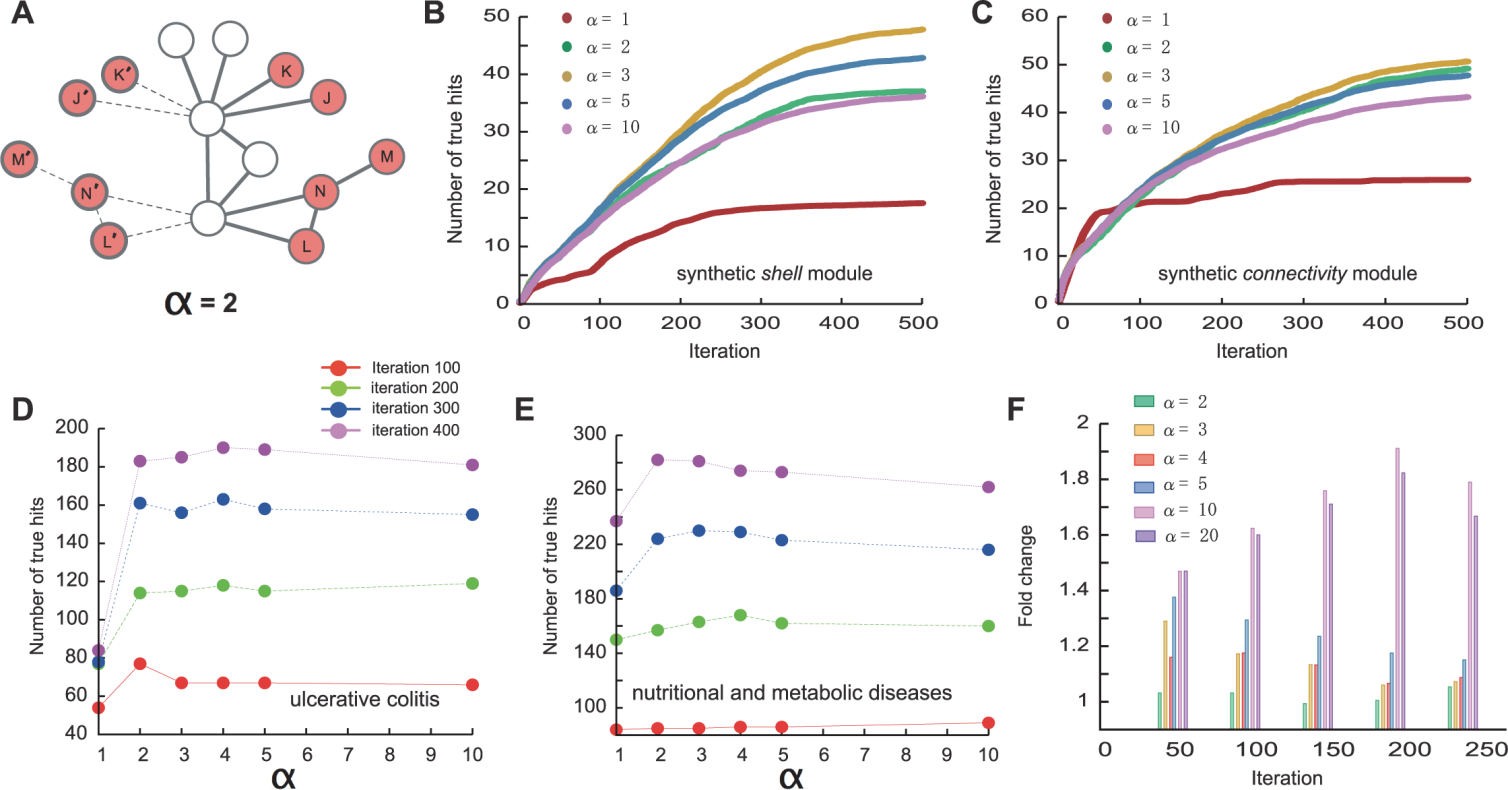
Extending the DIAMOnD algorithm. (A) Illustration of how the algorithm can be modified to give the initial seed proteins a higher weight α = 2 by (virtually) doubling the seed proteins while keeping their interactions. Tuning α results in different sets of detected proteins. (B,C) Comparing the performance for varying values of α in synthetic shells (B) and connectivity significance (C) modules, respectively. The best results are obtained for α = 3. (C) The performance may also saturate for α larger than a certain value. For a given disease α can be tuned to optimize the results. Performance of DIAMOnD with respect to different values of α is shown for ulcerative colitis (D) and nutritional and metabolic diseases (E). These plots suggest that at α = 2 the number of true positives is maximal. (F) Overall, α ∼10 results in the best performance of DIAMOnD across 70 diseases. The individual values may vary considerably, however, suggesting an individual optimization for best results.

The DIAMOnD methodology can be easily extended to incorporate weighted links or nodes. In the iteration process introduced above, the seed proteins are treated the same way as the predicted proteins agglomerated into the module at later iteration steps. We can, however, give higher weights to the seed proteins compared to those that are only predicted. This can be achieved by introducing an additional weight α> 1 for the seed proteins and α = 1 for all other proteins. By considering links to nodes with higher weights to be α times stronger, the direct neighbors of seed proteins have a higher chance of being identified. Technically, this is implemented by artificially increasing the number of seed genes, for example by duplicating their number in the case of α = 2, while maintaining their original interactions (Fig. 6A). The generalized form of Equation (1) then becomes:

$$p(k,k_{s},k_{s_{0}})=\frac{\left(\begin{array}{c} s+(\alpha -1){\text{s}}_{0}\\ k_{s}+(\alpha -1)k_{s_{0}} \end{array}\right)\left(\begin{array}{c} N-s\\ k-k_{s} \end{array}\right)}{\left(\begin{array}{c} N+(\alpha -1)k_{s}\\ k+(\alpha -1)k_{s}{}_{{}_{0}} \end{array}\right)}$$ (3)

By tuning α and comparing the different resulting DIAMOnD sets we can optimize their biological relevance. In synthetic modules, the recovery rate could thereby be increased 2 to 3 times in comparison to the original version of the algorithm for which the recovered fraction saturates (Fig. 6B,C). On the set of 70 diseases, the optimal values for α vary considerably (see Fig. 6D and 6E for the examples of *lysosomal storage diseases and ulcerative colitis*). Based on the pathway validations, we find that α ≈ 10 performs best for many diseases (Fig. 6F). As noted above, however, the validation according to pathways is biased towards immediate neighbors of the seed genes and we therefore expect that optimal values of α will depend on the specific application and the validation data that are used. We also observed that introducing α allows for the construction of larger modules by helping avoid plateaus in the identification of relevant proteins (Fig. 6B-E).

## Discussion

The hypothesis that disease associated proteins tend to interact with each other in the human Interactome underlies all network-based prioritization methods. Yet, for most diseases we found that only a relatively small fraction of known seed proteins in fact interact with each other. As a consequence, diseases cannot be associated with topologically dense network communities. Instead of the interaction *density*, we identified the interaction *significance* as the key quantity to characterize the connection patterns among disease proteins. While in principle this could be a consequence of our currently still very limited knowledge of disease associated proteins and their interactions, our results suggest that there is in fact a fundamental difference between disease modules and topological modules. Biologically, it is indeed plausible that disease modules do not necessarily coincide with densely interconnected topological modules. Highly interconnected proteins often represent functional units to perform a certain cellular task. Diseases, on the other hand, are likely to be the result of perturbations among several functional modules and therefore expected to span across functional modules/topological communities.

Our analysis of the connection patterns of known disease proteins further allowed us to design a predictive and robust algorithm to uncover unknown disease associations and construct a comprehensive disease module. For both synthetic test modules and real disease modules the recall of DIAMonD generally does not depend on the level of completeness in the initial set of seed proteins, but is rather a property of the module itself. This can be used to estimate the expected true positive rate in the predictions and is particularly convenient for predicting new disease associations, where the total number of proteins involved in a disease is not known. While the outcome of DIAMOnD does not depend sensitively on the exact set of seed proteins, there typically are a few crucial seed proteins whose omission leads to drastically different and presumably random results. These crucial proteins are characterized by their high degree. Their topological importance suggests also particularly important roles for the pathobiological mechanisms of the disease. Overall, the final disease modules typically consist of one large component that contains all DIAMOnD genes and 30%-60% of the initially disconnected seed proteins, the rest remaining disconnected. The integration of the several initially disconnected seed clusters into a broader disease module and the elucidation of the network paths that interconnect them is crucial for a holistic understanding of the pathobiology and molecular mechanisms underlying complex diseases. Whether the remaining disconnected seed proteins could be integrated if the Interactome data was more complete, or whether their disease associations are spurious remains an open question.

## Materials and Methods

### Interactome construction

We only consider direct physical protein interactions with reported experimental evidence. For this, we consolidated several data sources as described in [12]:

- Regulatory interactions: We used the TRANSFAC [38] database that lists regulatory interactions derived from the presence of a transcription factor binding site in the promoter region of a certain gene. The resulting network consists of 774 transcription factors and genes connected via 1,335 interactions.
- Binary interactions: We combine several yeast-two-hybrid high-throughput datasets [10,39–42] with binary interactions from IntAct [43] and MINT [44] databases. The sum of these data sources yields 28,653 interactions between 8,120 proteins.
- Literature curated interactions: These interactions, typically obtained by low throughput experiments, are manually curated from the literature. We use IntAct, MINT, BioGRID [45] and HPRD [46], resulting in 88,349 interactions between 11,798 proteins.
- Metabolic enzyme-coupled interactions: Two enzymes are assumed to be coupled if they share adjacent reactions in the KEGG and BIGG databases. In total, we use 5,325 such metabolic links between 921 enzymes from [47].
- Protein complexes: Protein complexes are single molecular units that integrate multiple gene products. The CORUM database [48] is a collection of mammalian complexes derived from a variety of experimental tools, from co-immunoprecipitation to co-sedimentation and ion exchange chromatography. In total, CORUM yields 2,837 complexes with 2,069 proteins connected by 31,276 links.
- Kinase network (kinase-substrate pairs): Protein kinases are important regulators in different biological processes, such as signal transduction. PhosphositePlus [49] provides a network of peptides that can be bound by kinases, yielding in total 6,066 interactions between 1,843 kinases and substrates.
- Signaling interactions: The dataset from [50] provides 32,706 interactions between 6,339 proteins that integrate several sources, both high-throughput and literature curation, into a directed network in which cellular signals are transmitted by proteins-protein interactions. Note that we do not take the direction of these interactions into account.

The union of all interactions from *(i)-(vii)* yields a network of 13,460 proteins that are interconnected by 141,296 physical interactions.

### Disease-gene associations

The corpus of 70 diseases was manually chosen by a medical expert, with the additional criteria of at least 20 associated genes reported in the literature. The gene-disease associations were retrieved from OMIM (Online Mendelian Inheritance in Man; http://www.ncbi.nlm.nih.gov/omim) [51] and GWAS (Genome-Wide Association Studies. The OMIM associations we use also include associations from UniProtKB/Swiss-Prot and have been compiled by [13]. The disease-gene associations from GWAS are obtained from the PheGenI database (Phenotype-Genotype Integrator; http://www.ncbi.nlm.nih.gov/gap/PheGenI) [9] that integrates various NCBI genomic databases. We use a genome-wide significance cutoff of *p*-value ≤ 5 · 10^–8^.

### Local modularity *R*

To quantify the extent to which disease proteins correspond to topological communities, we use the *local modularity R* [23]. The community character of a set of nodes *C* is determined by the “sharpness” of its boundary, i.e by how well it is separated from the rest of the network. The boundary *B* consists of all nodes in *C* that have connections to nodes outside the community (Fig. 1K). The local modularity *R* is then defined as the number of links attached to nodes in *B* that do not leave the community, normalized by their total number of links. This can be written as

$$R=\frac{\sum_{ij}B_{ij}\delta (i,j)}{\sum_{ij}B_{ij}}$$

where *B*_*ij*_ is the adjacency matrix of the boundary nodes and *δ*(*i,j*) = 1 if both nodes *i* and *j* are in *C*, otherwise *δ*(*i,j*) = 0.

The comparison with random control was done by selecting for each disease the same number of proteins at random from the Interactome (100 times). We then used a Kolmogorov-Smirnoff test to estimate the significance of the difference between the distribution of disease proteins and the respective distribution obtained in the randomization.

### Topological community detection methods

We use three well-established, methodologically distinct algorithms:

- A link community algorithm from [14], which provides a hierarchical clustering of all links in the network. We use the default cut-off at the optimal partition density.
- The parameter-free Louvain method [21], which maximizes the global modularity of the network.
- The Markov Cluster Algorithm (MCL) [24], which is based on random flow. We use the default settings (inflation parameter *r* = 2) of version mcl-12–068.

### Random walk based disease gene prioritization

We implemented a method from [35] that prioritizes candidate genes based on network diffusion. The seed genes serve as starting points for a random walker that wanders from node to node along the links of the network. At every time step of the iterative algorithm, the walker moves to a randomly selected neighbor of its current position. After every move the walker is reset to a randomly chosen seed gene with a given probability *r* (we use *r* = 0.4). After a sufficient number of iterations the frequency with which the nodes in the network are visited converges and can be used to rank the corresponding genes. Genes that are visited more often are considered to be closer to the seed genes and therefore more relevant to the disease than those who are visited less often.

### Network randomization

We use two models to construct ensembles of randomized networks with varying degrees of noise and incompleteness compared to the original Interactome:

- To investigate the effects of network incompleteness we construct *pruned networks* by removing a fraction of randomly selected links from the Interactome.
- To explore the impact of noise in the Interactome we use *partially rewired networks* in which a fraction of randomly selected links are split and then randomly reconnected. This procedure corresponds to the configuration model [52,53] and does not alter the degrees of the nodes, i.e. only the specific interaction partners of the nodes are randomized, not their overall number. Note that the original network is perturbed considerably even at small fractions of rewired links as both existing links are removed and simultaneously new ones are established.

### DIAMOnD implementation

The number of times we need to calculate the computationally relatively expensive *p*-values can be considerably reduced by noticing that two proteins with the same values of either *k*_*s*_ or *k* can be ranked directly according to their value in the respective other parameter, see Eqs. (1) and (2): If two proteins have the same degree *k*, the one with higher *k*_*s*_ will result in less terms in the sum in Eq. (2) and consequently a lower *p*-value. Similarly, between two proteins with the same number of connections to seeds *k*_*s*_, the one with lower *k* will result in lower *p*-value. This results in the following procedure: At each iteration step, we first classify the nodes based on their k_s_ and rank the node with lowest *k* highest within that class. Next, we classify the top ranks of each class by their degree *k* and choose the ones with highest *k*_*s*._ Finally, we calculate the exact *p*-value for the remaining nodes. This procedure guarantees that the number of candidate nodes will reduce to at most *s* nodes per iteration, as *k*_*s*_ cannot exceed *s* (note that *s*_*i*_ → *s*_*i*_+1 at each iteration). In the worst-case scenario, and without further reducing the candidate nodes by their degree *k*, we are left with *s* nodes for which we need to calculate *p*-values. Assuming we need to identify *N* nodes from the network, the time complexity of the algorithm is of the order *s*+(*s*+1)+…+(*N-1*)+*N* ∼ $\frac{N(N-1)}{2}=O\left(N^{2}\right)$. This compares favorably with other well established algorithms such as the random walk based method, whose complexity is between *O*(*N*log*N*) and *O*(*N* ^*3*^) [54,55].

### Topological validation, *N-1* analysis and persistence

We quantify the extent to which the removal of a seed node affects the outcome by two parameters: *(i)* the deviation from the original outcome and *(ii)* the persistence of that deviation for many iterations:

deviation=1−overlap

where the *overlap* is measured by the number of proteins that are in common between the original DIAMOnD outcome and the DIAMOnD outcome after the removal of seed genes. The *persistence* of a deviation is measured as

$$\text{Persistence=}\frac{\text{Total number of iteration steps where the deviation persists}}{\text{Total number of iterations}}$$

High persistence indicates that the removal of a node results in a deviation that holds across all iterations. However, typically we find that the perturbations introduced by removing a single seed node are compensated after a few iterations.

### Gene annotations

We use Gene Ontology (GO) for all genes are extracted from [http://www.geneontology.org/, downloaded Nov. 2011]. We only use high confidence annotations associated with the evidence codes EXP, IDA, IMP, IGI, IEP, ISS, ISA, ISM or ISO. In particular, we do not use annotations inferred from physical interactions (evidence code IPI) in order to avoid circularity. To obtain a complete set of GO terms from the reported most specific term for each gene, all annotations are propagated upwards on the full tree.

The pathway annotations are extracted from the Molecular Signatures Database (MSigDB) published by the Broad Institute, Version 3.1 [56]. MSigDB integrates several different pathway databases; we use the ones from KEGG, Biocarta and Reactome.

### Biological validation analysis

To validate the potential disease relevance of the predicted candidate genes (from either DIAMOnD or RW), we compare their biological characteristics to the ones of the initial seed genes using the following workflow:

- First we identify the set of GO terms (pathways) that are significantly enriched within the given set of seed genes using Fisher’s exact test (Bonferroni corrected *p*-value<0.5).
- For each candidate gene we then check whether it is annotated with any of these significant terms. Genes with common annotations are considered as true positives.
- We compare the performance of DIAMOnD genes to seed genes as well as to random expectation for the same number of genes drawn randomly from network. The performance is based on the number of candidate genes that are considered true positives. To quantify the statistical significance of a given number of true positives at a given iteration step we use a sliding window approach: At each iteration step *i*, we consider the same number of candidate genes as there are seed genes for the respective disease. If there are 100 seed genes, for example, we use the genes in the interval [i-100/2, i+100/2] and count the number true positives among these genes. The statistical significance of an observed number is then determined using Fisher’s exact test. Matching the number of candidate genes with the number of seed genes allows us to compensate for the dependence of *p*-values on the underlying set size, thereby enabling us to directly compare DIAMOnD sets at different iteration steps, as well as DIAMOnD genes and seed genes.

## Supporting Information

S1 Fig Size distribution of the topological communities in the Interactome as identified by (A) link clustering, (B) the Louvin method and (C) the MCL method. (D-F) Number of community-disease pairs with significant overlap vs. their *Jaccard* similarity *J* for the three methods. No identified topological community coincides (*J* = 1) with a full set of disease genes.
(EPS)

S2 Fig Properties of the synthetic *Shell* modules. (A) Illustration of the construction process: An initial node is selected at random and all first and second neighbors are added to the module. The exact topological properties of the resulting modules depend on the initial node. Panel (B) shows how the synthetic module size varies with the degree of the initial node.
(EPS)

S1 Data Annotated Interactome data. (TSV)

S2 Data Disease gene association data for 70 diseases. (TSV)

S1 Code A python implementation of the DIAMOnD algorithm. (PY)
